# iTDtest: an Easy-to-Handle and Visual Assay To Detect Tolerant and Persister Cells in an Antibiotic Combination Regimen

**DOI:** 10.1128/mbio.00362-22

**Published:** 2022-06-13

**Authors:** Laurence Van Melderen

**Affiliations:** a Cellular and Molecular Microbiology–CM2, Department of Molecular Biology, Université Libre de Bruxelles, Brussels, Belgium

**Keywords:** antibiotics, persistence, tolerance

## Abstract

In a recent article, Balaban and colleagues developed the iTDtest allowing characterization of the type of interactions between different antibiotics at bactericidal concentrations (J.-F. Liu et al., mBio 13:e00004-22, 2022). This visual and semiquantitative assay is designed to determine how antibiotic cocktails affect tolerance and persistence, two phenomena of major importance for the eradication of difficult-to-treat pathogens. Importantly, Balaban and colleagues identified antibiotic combinations allowing for complete clearance of persister and tolerant cells. This commentary discusses the translation of this assay in clinical settings, where antibiotic combination therapies appear to be applied in specific contexts, such as in acute infections or in the case of multidrug or extensively drug-resistant pathogens.

## COMMENTARY

## COMMENTARY

The antibiotic resistance crisis is rising worldwide and is announced as one of the next pandemics by the World Health Organization (WHO). By 2050, multidrug-resistant bacteria could cause the death of 10 million people each year. Even more alarming, the WHO warns that the pipeline for new antibiotics discovery and development is drying up with only a few leads under clinical trials.

**FIG 1:**
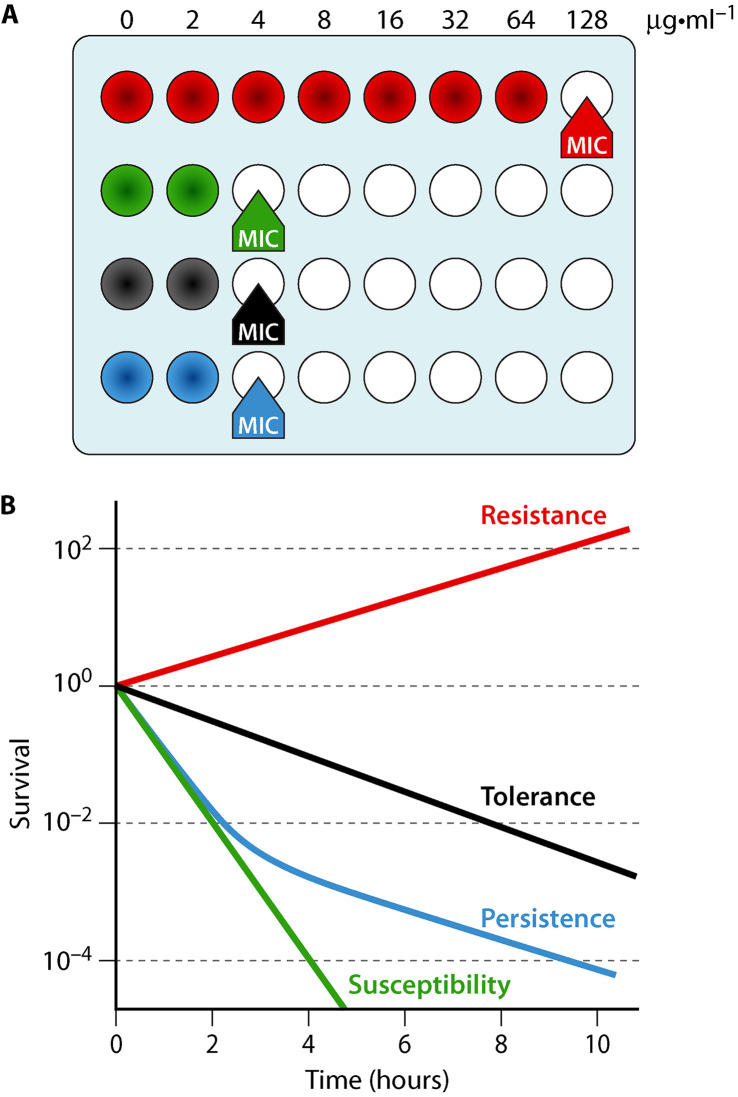
Resistance, susceptibility, tolerance, and persistence to antibiotics. (A) The MIC to an antibiotic is higher for a resistant (red) than for a susceptible (green) bacterial strain. MICs are similar for tolerant strains or strains producing persisters (blue) and a susceptible strain. Colored wells show bacterial growth. (B) Classical bacterial survival curves over time during antibiotic exposure. Resistant strains grow in the presence of the antibiotic (red). Susceptible strains exhibit complete killing in the presence of the antibiotic (green). Tolerance is characterized by the ability of a strain to survive transient high concentration antibiotic exposure (black). Persistence is characterized by a biphasic curve: the first part representing killing of susceptible cells, the second part indicative of persister cells that survive prolonged antibiotic exposure (blue). Concentrations and timescales are for illustration purposes. (Modeled based on reference 2.)

In addition to resistance, antibiotic tolerance and persistence are increasingly recognized as major players in the relapse of infections and emergence of antibiotic resistance (1). In contrast to resistant cells, tolerant and persistent cells do not divide during antibiotic treatment, are sensitive to the antibiotics i.e., do not exhibit higher MIC in contrast to resistant cells (Fig. 1), and do not carry genetic modifications (2). Persister cells constitute small subpopulations of tolerant cells arising at a very low frequency within the population (Fig. 1). Upon antibiotic removal, tolerant and persister cells resume growth and give rise to a new population, as sensitive to the antibiotic as the initial population.

**FIG 2:**
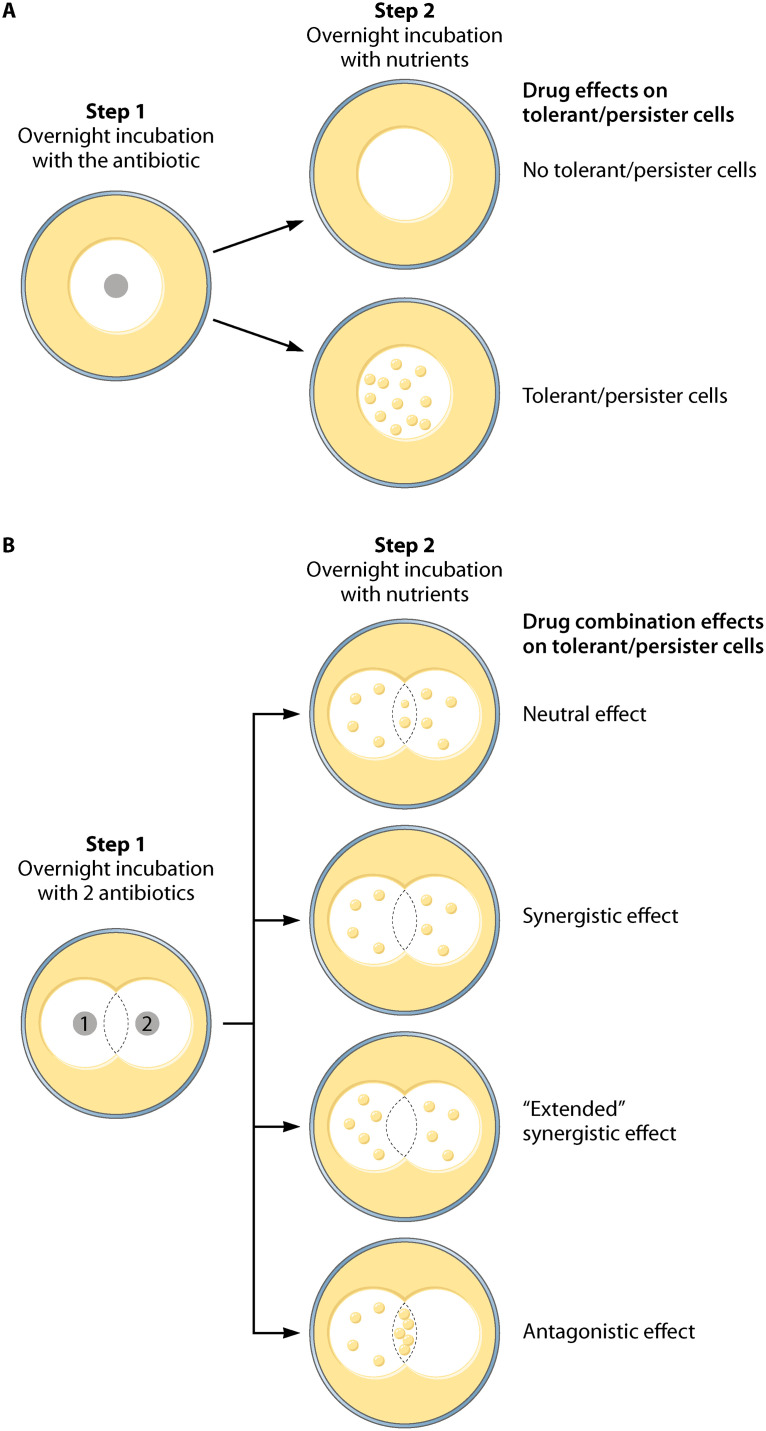
TDtest and iTDtest. (A) The TDtest comprises two steps: bacteria are plated on solid medium and the antibiotic containing disk (gray circle) is placed on the plate (large outer circle). After a first overnight incubation, the antibiotic containing disk is replaced by a disk containing nutriments. Plates are incubated overnight to allow the regrowth of tolerant and persistent cells (small yellow dots) present in the growth inhibition zone (white inner circle) that have survived the antibiotic exposure. (B) The iTDtest combines different antibiotics (gray circles 1 and 2) on the same plate and follows the same procedure as described for panel A. Different outcomes are observed depending on the nature of the interactions between the two antibiotics (at the intersection of the two antibiotic inhibition zones). Neutral interaction is revealed by similar regrowth of tolerant/persister cells in the intersection zone compared to that of each antibiotic. Synergistic effect is revealed by the clearance of tolerant/persister cells in the intersection zone. “Extended” synergy is described by the extension of the intersection zone toward one of the antibiotics. An antagonistic effect is revealed by an increased number of regrown tolerant/persister cells in the intersection zone (antibiotic 1 being antagonistic on antibiotic 2).

Classically, the antibiotic tolerance and/or persistence ability of a given strain is evaluated by performing liquid time/kill curve assays (Fig. 1). This assay measures the capacity of a bacterial culture treated with an antibiotic at a concentration above the MIC to form colonies on solid medium plates without the antibiotic as a function of duration of the treatment. The feasibility of these assays to test a panel of different antibiotics at different concentrations for different durations is limited in the routine clinic settings. In 2017, the group of Nathalie Balaban developed an easy and semiquantitative method to detect persistent and tolerant cells (Fig. 2A) (3). The TDtest for Tolerance Disk Test is based on the classical MIC-determination Kirby-Bauer disk assay (4) and relies on the addition of nutrients (generally carbon source and amino acids) after a first overnight incubation with the antibiotic. This extra step allows the regrowth of tolerant and persistent cells present in the growth inhibition zone that have survived the antibiotic exposure. Note that the classical Kirby-Bauer disk assay does not permit the detection of tolerant and persister cells as their MIC remain unchanged. In their recent paper, Balaban and colleagues further implemented the TDtest to measure the interactions between several antibiotics (iTDtest for Interaction Tolerance Disk test) (Fig. 2B) (5). This assay consists of placing two disks containing different antibiotics at a distance, allowing observation of how bacteria behave at the intersection region where they are submitted to the action of both antibiotics compared to the external regions where they are in contact with only one of the antibiotics. As in the TDtest, after overnight exposure to the antibiotics, nutrients are added to allow the growth of tolerant and/or persister cells. This assay allows the determination of synergistic and antagonistic drug interactions on tolerant and/or persister cells. It is a promising alternative to the checkerboard array method that is classically used to test the interactions between two antibiotics. This method is adapted from the standard broth-based MIC determination test and allows measurement of the fractional inhibition concentration (FIC) index, reflecting the effect of a combination of two antibiotics on the growth rate (6). While the checkerboard array method proved to be valuable for determining whether two antibiotics are either synergistic or antagonist at concentrations near the MIC, it does not provide information regarding the interaction types at high and bactericidal concentrations. This is of major importance since in a clinical setup, high antibiotic concentrations are commonly used, and interactions might be different at these concentrations compared to near-MIC ones. iTDtest data combining different antibiotics on the wild-type and high tolerant mutant strains were consistent with time/kill assays, thereby validating the iTDtest. In addition to being easy to handle, semiquantitative, and detecting drug synergy or antagonism at high bactericidal doses, the iTDtest allows for testing higher-order combinations by using up to three disks containing different antibiotics on the same plate or by impregnating a single disk with several antibiotics. Moreover, iTDtest can predict directional interactions, i.e., beneficial effect of treating with a second antibiotic in addition to the basic antibiotic treatment. Balaban and colleagues tested different antibiotic combinations on wild-type and tolerant Escherichia coli strains. While confirming that ampicillin and rifampicin are strongly antagonist, these researchers bring to light a synergistic effect between ampicillin and kanamycin or streptomycin on the appearance of persister cells. Importantly, adding kanamycin or streptomycin both at high and sub-MICs reduces persistence to ampicillin. Finally, they showed that combining ampicillin, rifampicin and kanamycin completely eradicate persisters formed by an E. coli antibiotic tolerant strain.

Given that antibacterial drug discovery is a slow process and that the current clinical pipeline is mostly limited to derivatives of classical antibiotic molecules, the short-term approach to combat recalcitrant bacteria might rely on the use of antibiotic combinations (7, 8). However, it does not appear to be in common practice, with only scarce examples being reported in the literature, notably in the case of acute infections—before the etiological bacterial pathogen is identified, in polymicrobial infections, in Mycobacterium tuberculosis infections, or in the case of febrile neutropenic patients (9). With the emergence of multidrug-resistant or extensively drug-resistant pathogens such as some Acinetobacter baumannii, Pseudomonas aeruginosa, Klebsiella pneumoniae, or Escherichia coli strains, antibiotic cocktails have regained interest notably with combination regimens comprising colistin, tigecycline, aminoglycosides, vancomycin, and/or carbapenem, depending on the pathogen (10, 11). Antibiotic combination with a synergistic effect should theoretically reduce the risk of therapeutic failure and the emergence of antibiotic tolerance and resistance (12). However, establishing a “universal” synergetic combination might prove to be difficult since different isolates from a given pathogen do not necessarily show comparable combination sensitivity, highlighting the need to test on a case-by-case basis the synergistic potential of antibiotic combinations (see, notably, references 13 and 14). In this context, the iTDtest developed by Balaban and colleagues might be very useful to implement in clinical microbiology laboratories.
